# Metabolic programming of a beige adipocyte phenotype by genistein

**DOI:** 10.1002/mnfr.201600574

**Published:** 2016-12-06

**Authors:** Sadat A. Aziz, Luisa A. Wakeling, Satomi Miwa, Goiuri Alberdi, John E. Hesketh, Dianne Ford

**Affiliations:** ^1^ Institute for Cell and Molecular BiosciencesNewcastle upon TyneUK; ^2^ School of Dental SciencesNewcastle UniversityNewcastle upon TyneUK; ^3^ Department of Obstetrics and GynaecologyUniversity College DublinDublinUK; ^4^ Faculty of Health and Life SciencesNorthumbria UniversityNewcastle upon TyneUK

**Keywords:** Brown adipose tissue, Estrogen receptor, Genistein, SIRT1, White adipose tissue

## Abstract

**Scope:**

Promoting the development of brown or beige adipose tissue may protect against obesity and related metabolic features, and potentially underlies protective effects of genistein in mice.

**Methods and results:**

We observed that application of genistein to 3T3‐L1 adipocytes changed the lipid distribution from large droplets to a multilocular distribution, reduced mRNAs indicative of white adipocytes (ACC, Fasn, Fabp4, HSL, chemerin, and resistin) and increased mRNAs that are a characteristic feature of brown/beige adipocytes (CD‐137 and UCP1). Transcripts with a role in adipocyte differentiation (Cebpβ, Pgc1α, Sirt1) peaked at different times after application of genistein. These responses were not affected by the estrogen receptor (ER) antagonist fulvestrant, revealing that this action of genistein is not through the classical ER pathway. The Sirt1 inhibitor Ex‐527 curtailed the genistein‐mediated increase in UCP1 and Cebpβ mRNA, revealing a role for Sirt1 in mediating the effect. Baseline oxygen consumption and the proportional contribution of proton leak to maximal respiratory capacity was greater for cells exposed to genistein, demonstrating greater mitochondrial uncoupling.

**Conclusions:**

We conclude that genistein acts directly on adipocytes or on adipocyte progenitor cells to programme the cells metabolically to adopt features of beige adipocytes. Thus, this natural dietary agent may protect against obesity and related metabolic disease.

## 1 Introduction

Electron transport by the mitochondrial respiratory chain generates a proton gradient that is used to synthesise ATP. In the mitochondria of brown adipose tissue (BAT) this function is uncoupled from ATP synthesis by the action of uncoupling protein 1 (Ucp1/UCP1), hence dissipating energy through nonshivering thermogenesis. Transgenic mouse models show that BAT protects against diet‐induced obesity, insulin resistance and type 2 diabetes 1. Radionuclide labelling studies show that BAT stimulated by cold acclimation is detectable in some human adults 2 and was associated with a healthier metabolic phenotype including lower BMI, fat mass, blood glucose, and cholesterol 3, 4. Differences in the adipokine profile of brown versus white adipocytes are likely important drivers of many of these effects. There is also a compelling argument that promoting the development of BAT could be a strategy to maximize the benefits of a low protein, high carbohydrate diets. Careful analysis of studies in rodents and insects shows that such diets robustly promote longevity, and observational studies in human support this notion. However the diets with this composition often drive increased food intake and hence a gain in body fat 5. Promoting the development and/or activity of BAT is thus a potential approach to curb obesity and associated metabolic symptoms.

Cells that have features of brown adipocytes, including small mutilocular lipid droplets and higher Ucp1 expression associated with mitochondrial uncoupling, have been observed in depots of white adipose tissue (WAT). For example, cold exposure induced adipocytes that expressed Ucp1 in most depots of WAT in mice 6, 7, 8. Terms including brite (brown in white) or beige adipocytes are used to describe these cells. Beige and brown adipocytes appear to be distinguished by several features. Beige adipocytes express Ucp1 at high levels only in response to stimulation by factors that include PPAR‐ɣ and β‐adrenergic agonists. Also, the two different cell types arise from different precursors. It appears that most brown adipocytes, like skeletal muscle cells, derive from Myf5‐expressing mesodermal precursors, whereas it has been shown that beige adipocytes originate from precursors with no history of Myf5 expression, indicative of a common lineage with white adipocytes. However, this polarity in origin between brown and beige adipocytes is not absolute, since white adipocytes derived from precursors that express Myf5 have been observed in some adipose tissue depots 9, 10.

Beige adipocytes in WAT most likely result from differentiation of precursors 11. However, the possibility of transdifferentiation cannot be excluded. The precursors from which white and beige adipocytes originate appear distinct. The expression of the markers Cd137 is among features that distinguish beige precursors from those of white adipocytes 12. In humans multipotent precursor cells with the capacity to differentiate into cells with features of beige adipocytes have been isolated from adult human subcutaneous WAT, including abdominal fat 13. Thus, there is good support for the idea that dietary/pharmaceutical agents could induce the appearance of beige adipocytes in human WAT depots and hence confer metabolic benefit.

The isoflavone genistein is a plant polyphenol. Soyabeans are a particularly rich dietary source of genistein. The compound is similar structurally to the mammalian hormone 17‐β estradiol; thus it belongs to the group of compounds termed phytoestrogens. A large body of research on genistein, soya derivatives (in particular soya protein) and on soya‐based foods has uncovered evidence of potential beneficial actions against a range of conditions. The evidence for beneficial effects on cardiovascular health and protection against cancer 14 is substantial whereas the evidence that genistein can influence fat deposition and other (non‐cardiovascular) features of metabolic health is less robust. However observations made in rodents show variously that genistein alone or as a crude extract with other polyphenols can have an effect on energy expenditure, UCP expression and protect against the obesogenic effect of a high calorie diet 15, 16, 17, 18, 19, 20. We hypothesised that genistein affects white adipocyte phenotype to promote beneficial changes in physiology. We thus studied effects of genistein on cultured white adipocytes using the well‐characterized 3T3‐L1 cell line model.

## 2 Materials and methods

### 2.1 Cell culture and treatment

All reagents for cell culture and treatment were purchased from Sigma. 3T3‐L1 cells were grown in pre‐adipocyte medium (DMEM, 10% calf serum and 1% pencillin streptomycin) in 6‐well plates (for extraction of RNA) or in Seahorse V7 cell plates (for measurement of mitochondrial function). Forty eight hours after cells reached confluence, taken as day 0, cells were induced to differentiate by using pre‐adipocyte medium supplemented with 500 μM 3‐isobutyl‐1 methylxanthine (IBMX), 10μg/mL insulin, 250 nM dexmethasone, 8 μg/mL biotin, and 4 μg/mL pantothenic acid. After a further 48 h, this medium was replaced with pre‐adipocyte medium fortified with 10 μg/mL insulin, 8 μg/mL biotin, and 4 μg/mL pantothenic acid and cells were incubated for a further 48 h. Thereafter, cells were maintained in mature adipocyte medium (0.2 μg/mL insulin, 8 μg/mL biotin, and 4 μg/mL pantothenic acid), which was replaced every 2 days until the end of the experiment. Normally lipid droplets began to appear 5–6 days after inducing differentiation. Genistein and fulvestrant were added as 100× solutions in DMSO at the point where differentiation was induced and an equal volume of DMSO was added to control cells. These media were replaced every 2 days.

For staining with Oil Red O cells were washed with warm PBS, fixed with 10% neutral formaldehyde for 2–3 h at room temperature then rinsed quickly with 60% isopropanol and allowed to dry. Cells were then overlaid with Oil Red O (Sigma; 0.5% w/v diluted 3:2 in double distilled water) and incubated for 2–3 h at room temperature before washing 3–4 times with double distilled water and photographing under a light microscope.

### 2.2 RNA extraction and RT‐qPCR

Total RNA was extracted using TRIzol (Life Technologies) then the PureLink^TM^ RNA Mini Kit (Life Technologies), following the manufacturer's protocol. Reverse transcription was carried out using Superscript™ III Reverse Transcriptase (Life Technologies) according to the manufacturer's protocol and using a 20 μL reaction volume containing 0.5‐1 μg RNA with 150 ng random primers (Promega), 10 μM dNTPs (Bioline), and 40 U RNase Inhibitor (Promega).

PCR reactions were performed in 10 μL volumes using SYBR Green I Master Mix (Roche) with each primer (Table 1) at a concentration of 0.05 μM. Cycling parameters were 95˚C for 5 min, followed by 45–55 cycles of 95˚C for10 s, 55–60˚C for 10 s, 72˚C for 15 s. Relative transcript quantities were calculated using the 2^∆∆Ct^ method using *Top1* and *NONO* as reference genes.

**Table 1:** Primers and thermal cycling parameters used for RT‐qPCR. Sequences are in the 5’‐3’ direction and subscript numerals refer to positions within the sequences deposited under the stated Genbank accession numbers. Forward (5’) then reverse (3’) primer sequences are stated in each case

Gene	Transcript Genbank accession number	Primer sequences	Anneal temp. (°C)	Holding times (at 95°C; annealing temperature; 72°C)	Number of cycles
*Acaca*	NM_133360.2	2587ATGTCCTGGATAACCTGGTC2606 2710GATATCCTGCAGCTCTAGCA2691	55	10 s; 5 s; 10 s	45
*Fasn*	NM_007988.3	615GTGGACATGGTCACAGATG634 770CATAGCTGACTTCCAACAGC751	55	10 s; 5 s; 10 s	45
*Fabp4*	NM_024406.2	297GACAGGAAGGTGAAGAGCAT316 411GCCTTTCATAACACATTCCAC391	55	10 s; 5 s; 10 s	45
*Lipe*	NM_010719.5	1951GTCAGTGCCTATTCAGGGAC1970 2071AGTTGAGCCATGAGGAGGC2053	55	10 s; 5 s; 10 s	45
*Rarres2*	NM_027852.2	231CTGTGCAGTTGGCCTTCCAAG251 359GGTTTGATTGTGCACTCCGG340	55	10 s; 5 s; 10 s	45
*Retn*	NM_022984.4	38CAGAACTGAGTTGTGTCCTGC58 165CTTGTCGATGGCTTCATCGATG144	55	10 s; 5 s; 10 s	45
*Sirt1*	NM_019812.3	777GCTGTGAAGTTACTGCAGG795 874GCAAGGCGAGCATAGATAC856	56	10 s; 5 s; 10 s	45
*Cebpb*	NM_009883.3	830CAAGGCCAAGATGCGCAAC848 944GCAGCTGCTTGAACAAGTTC925	56	10 s; 5 s; 10 s	45
*Ppargc1a*	NM_008904.2	2151CAATTGAAGAGCGCCGTGTG2168 2296GTCACAGGTGTAACGGTAGGTG2275	55	10 s; 5 s; 10 s	45
*Ucp1*	NM_009463.3	563CCTGCCTCTCTCGGAAACAA582 618TGTAGGCTGCCCAATGAACA599	60	10 s; 5 s; 10 s	55
*Tnfrsf9*	NM_011612.2	160TCATTGTGCTGCTGCTAGTGG180 316CTGCACACACTCTGCAGATGT296	55	10 s; 5 s; 10 s	45
*Nono*	NM_023144.2	1396TGCTCCTGTGCCACCTGGTACTC1418 1544CCGGAGCTGGACGGTTGAATGC1523	55	10 s; 5 s; 10 s	45
*TOP1*	NM_003286.2	Human TOP1 Primer Mix (HK‐SY‐hu‐1200; Primer Design)	60	10 s; 5 s; 10 s	45

### 2.3 Measurement of mitochondrial function

Mitochondrial function was measured using the Seahorse XF‐24 analyser (Seahorse Bioscience). Measurements were made on day 12 after inducing differentiation. The cartridge was hydrated with calibration buffer (Seahorse Bioscience) in a non‐CO_2_ incubator at 37°C for approximately 4–5 h before use. Solutions for injection were prepared in the assay medium, which was DMEM (Sigma, D‐5030) with 5 mM D‐glucose (Sigma), 2% L‐Glutamax (Life Technologies), 3% calf serum (Sigma), 1% sodium pyruvate (Life Technologies). Cells were incubated in assay medium in a non‐CO_2_ incubator at 37°C for 1 h before use. The following drugs were injected sequentially: oligomycin (1 μg/mL), TTNPN (10 μM), FCCP (2.5 μM), antimycin A (2.5 μM) (all from Sigma). Proton leak was calculated as the difference in oxygen consumption rate (OCR) between cells after the addition of oligomycin and after the addition of antimycin. Maximum mitochondrial oxygen consumption was calculated as the difference in OCR between cells after the addition of FCCP and after the addition of antimycin. Cell lysates were prepared from each well at the end of the experiment for the measurement of total protein content. Cells were suspended in a mixture of 1X PBS and protease inhibitor cocktail (Roche) (1 mL/well) using a scraper then centrifuged at 13 000 × *g* for 15 min at 4°C. The supernatant fluid was discarded and cell pellets were resuspended in 50 μL of RIPA lysis buffer (Sigma) plus protease inhibitor cocktail (Roche) then frozen at −80°C. Lysates were thawed and then centrifuged at 13 000 × *g* for 20 min to remove cell debris. Protein concentration was measured using Bradford reagent (Bio‐Rad) against a standard curve constructed using BSA. The measures of protein content were used to normalize the data.

### 2.4 Western blotting

Cell lysates were prepared and protein concentration was measured as described above. SDS‐PAGE, transfer of proteins to PVDF membrane and probing with primary and secondary antibodies to detect Sirt1 and α‐tubulin were as described previously 21. Signals for Sirt1 and α‐tubulin were measured using Odyssey software, as described previously 21, and data were expressed as a ratio, which was then normalized to control.

## 3 Results

**Figure 1:**
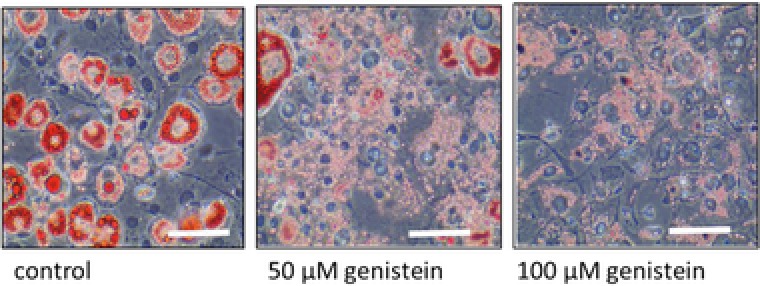
3T3‐L1 cells on day 12 after induction of differentiation to adipocytes in the presence of genistein at the concentrations indicated stained using oil red O to reveal the distribution of intracellular lipid. Scale bar 100 μM.

We tested the effect of genistein on adipoytes using the well‐established 3T3‐L1 cell line model, which is derived from mouse fibroblasts and can be stimulated to differentiate to acquire phenotypic features of white adipocytes 22, 23. We added genistein to the culture medium at concentrations of 10, 50, or 100 μM from the point of inducing differentiation. Compared with cells grown in parallel without the addition of genistein we observed a change in appearance that was induced more rapidly by higher concentrations of genistein. Staining with the lipid‐specific agent Oil Red O revealed a redistribution of lipid from large droplets to a multilocular pattern. Typical images are shown in Fig. 1. This pattern of lipid accumulation is a characteristic of brown/beige adipocytes.

**Figure 2:**
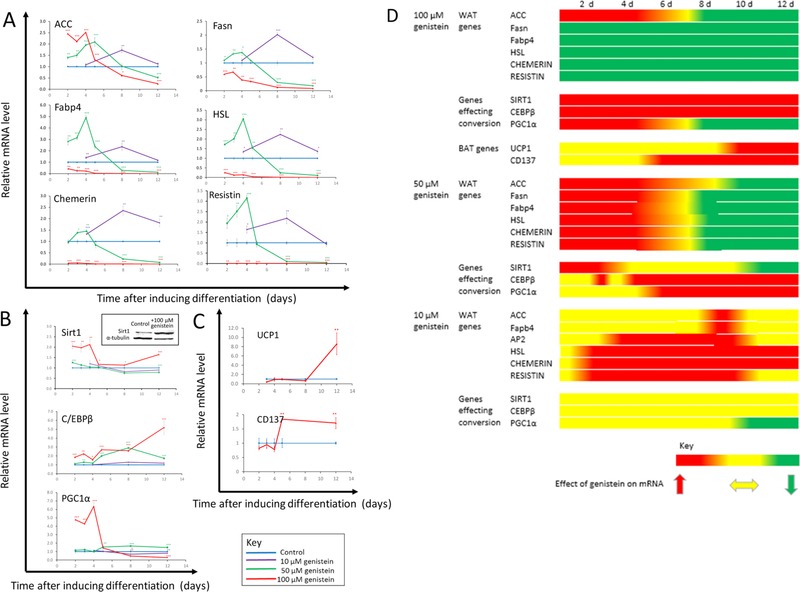
The effect of genistein (10, 50, or 100 μM) in 3T3‐L1 cells on mRNAs corresponding to genes expressed at high levels in white adipocytes (Panel A), genes that play a role in stimulating the development of brown/beige adipocytes (Panel B), or genes expressed preferentially in brown/beige compared with white adipocytes (Panel C). Genistein was applied on induction of differentiation (d 0) and measurements were made at d 12. Data are expressed relative to the reference genes *Topo1* and *NONO* and normalised to control. Values are mean ± SEM for *n* = 3–15 (derived from 3 replicates (wells) from 1 to 5 independent experiments). Statistical analysis was by one‐way ANOVA followed by Dunnett's test. *p ≤ 0.05, **p ≤ 0.01, ***p ≤ 0.001. The inset panel in panel B shows a representative western blot of protein extracted from cells at day 10 of treatment with 100 μM genistein and from control cells probed with antibodies against Sirt1 and α‐tubulin. Panel D provides an overview of the pattern of responses of these different categories of genes in the form of a heat map, where red shading indicates an increased quantity of mRNA, yellow shading indicates no difference from control and green shading indicates a reduced quantity of mRNA.

We next determined if genistein induced a shift in gene expression profile consistent with adipocyte browning. One response consistent with this action would be a reduction in mRNAs corresponding to genes expressed at high levels specifically in white adipocytes in cells treated with genistein. We selected *AcacaI* (acetyl‐CoA carboxylase), *Fasn* (fatty acid synthase)*, Fabp4* (fatty acid binding protein 4), *Lipe* (hormone sensitive lipase E)*, Rarres2* (retinoic acid receptor responder, also known as chemerin) and *Rten* (resistin) as a test panel of genes expressed at high levels in white adipocytes and measured the corresponding mRNAs (which we refer to as ACC, Fasn, Fabp4, HSL, chemerin and resistin, respectively) by RT‐qPCR. The time‐ and concentration‐dependence of the effects measured (Fig. 2) were consistent with a model where genistein initially promoted differentiation to white adipocytes but subsequently induced browning. At 100 μM genistein, the highest concentration tested, the expression of all of these genes was repressed at day 2, the first point of measurement, whereas at 50 μM gensitein there was an initial increase in this mRNA, which reached a maximum at day 4, then fell to levels lower than control by day 8. At the lower concentration of 10 μM, the maximum increase above control of all of these transcripts induced by genistein was observed on day 8 and no reduction in these transcripts compared with control had been achieved by day 12.

A second response to genistein consistent with induction of adipocyte browning would be an increase in the expression of genes that play a role in stimulating the development of brown/beige adipocytes. We selected *Cebpβ, Ppargc1α* and *Sirt1* as a panel of genes for which this function has been attributed. Cebpβ has been shown to mediate conversion of white to brown/beige adipocytes 1, 24, and deacetylation and thus activation of PPAR‐ɣ by Sirt1 caused brown remodeling of white adipose tissue 25. We compared levels of mRNAs corresponding to these 3 genes in cells treated with genistein with controls. Genistein caused an increase in expression of all three markers although the genes differed individually with respect to the time course over which expression peaked (Fig. 2). This pattern of expression may reflect action at different points in the differentiation process. Peaks in the expression of these genes that preceded the point at which cells appeared to have undergone full conversion to the beige adipocyte phenotype (i.e. 12 days after the introduction of genistein) are consistent with these genes playing in role in differentiation process. Cebpβ mRNA was increased by 100 μM genistein and, generally to a smaller extent, by 50 μM genistein progressively throughout the 12 day time course. At 100 μM genistein Pgc1α mRNA reached a peak at day 4 and had fallen to below levels in control cells by day 12, whereas at 50 μM genistein the increase was more moderate and reached a plateau over days 5–12. Sirt1 was induced substantially by only 100 μM genistein, and not by the lower concentrations tested, in a profile that comprised an early initial peak (days 2–4) followed by sustained more moderately enhanced expression. We also measured Sirt1 protein at day 10 of exposure to 100 μM genistein and observed an increase of approximately 12‐fold over control (*p* < 0.001 by Student's unpaired *t*‐test), according to quantification by densitometry of signals on western blots using protein from 2 independent experiments and measuring α‐tubulin as a loading control (Fig. 2; panel B, inset). Thus, despite the response at the mRNA level being more modest than for the CEPP and PGC1a, an increase in Sirt1 expression remains a likely important response to genistein associated with its ability to induce adipocyte browning.

To further determine if the reduced expression of genes characteristic of white adipocytes and the increases in expression of genes associated with promoting brown/beige adipocyte differentiation achieved at the higher concentration of genistein indicated a switch towards a brown/beige adipocyte phenotype we measured the parallel effect on Ucp1 and CD137 (gene *Tnfrsf91*) mRNAs, both of which are expressed preferentially in brown/beige compared with white adipocytes 12. At a concentration of 100 μM, genistein induced a large (8‐fold) increase in Ucp1 mRNA at day 12 and also a sustained higher level (∼2 fold) of CD137 mRNA over days 5–12 (Fig. 2). This large increase in Ucp1 mRNA in particular is very robust evidence of the cells having the characteristics of beige, rather than white, adipocytes since the protein product is the major functional mediator of the respiratory uncoupling that is core to the distinctive phenotype of brown or beige adipose tissue.

**Figure 3:**
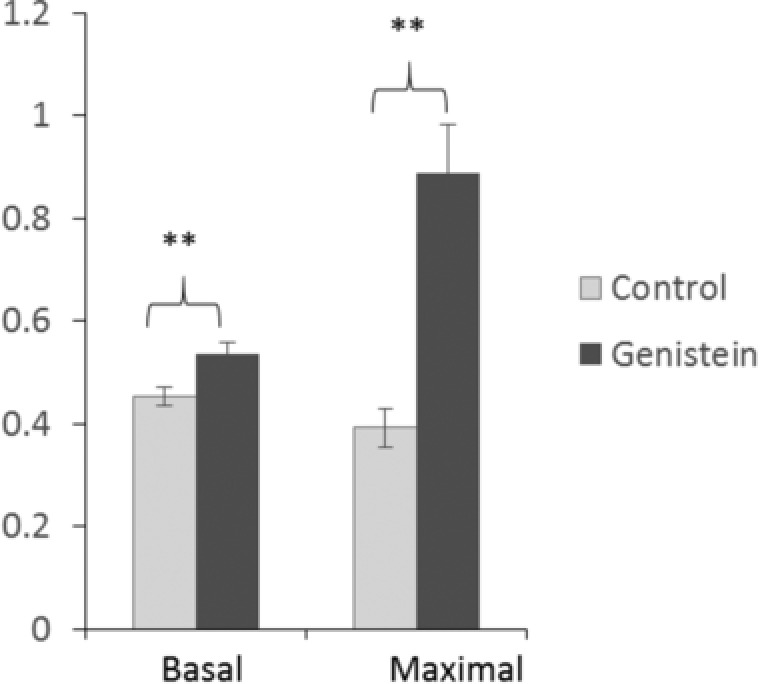
The effect of genistein on the proportional contribution of proton leak to basal and maximal respiratory capacity in 3T3‐L1 cells. The oxygen consumption rate by cells was measured on day 12 after induction of differentiation under control conditions or in the presence of 100 μM genistein. Data are from *n* = 10 wells for each condition. Statistical analysis was by Student's *t*‐test. **p ≤ 0.01.

To determine if the change in appearance and gene expression profile of the cells was associated with effects on mitochondrial function, specifically mitochondrial uncoupling, we measured the oxygen consumption rate at day 12 of treatment with genistein at baseline and after the sequential addition of oligomycin (to block ATP synthesis), TTNBP (a retinoic acid analogue that activates UCP1), FCCP (respiratory chain uncoupler) and antimycin A (inhibitor of respiratory chain complex III). Genistein increased baseline oxygen consumption, and the proportional contribution of proton leak to both basal and maximal respiratory capacity was greater for cells exposed to genistein than for controls (Fig. 3), revealing greater mitochondrial uncoupling in cells grown in the presence of genistein and hence consistent with the characteristics of beige adipocytes.

**Figure 4:**
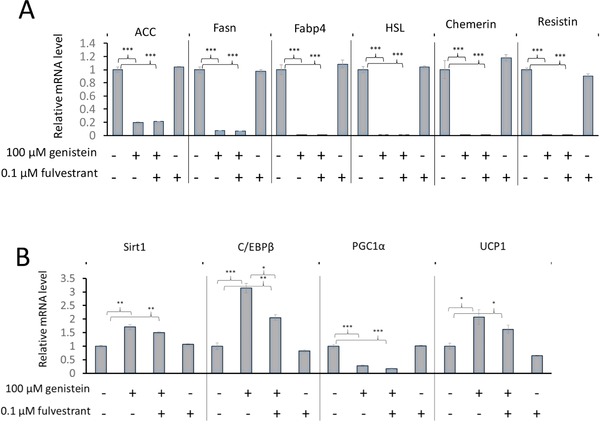
The effect in 3T3‐L1 cells of the estrogen receptor antagonist fulvestrant on responses to genistein of mRNAs corresponding to genes expressed at high levels in white adipocytes (Panel A) or genes that play a role in stimulating the development of brown/beige adipocytes or that are expressed preferentially in brown/beige compared with white adipocytes (Panel B). Genistein (100 μM), with or without fulvestrant (0.1 μM), as indicated, was applied on induction of differentiation (day 0) and measurements were made on day 12. Data are expressed relative to the reference genes *Topo1* and *NONO* and normalised to control. Values are mean ± SEM for *n* = 3. Statistical analysis was by one‐way ANOVA followed by Bonferroni’ multiple comparisons test. *p ≤ 0.05, **p ≤ 0.01, ***p ≤ 0.001.

On the basis that many reported cellular actions of genistein appear to be mediated through estrogen receptors (ERs), and based on evidence that genistein is an ER ligand 26, 27, we hypothesised that the effects of genistein we observed were mediated through binding to the ER. We tested this hypothesis by adding 100 μM genistein to the medium used to induce differentiation in the presence and absence of the ER antagonist fulvestrant. With the exception of the response of C/EBPβ, the presence of fulvestrant in the medium did not affect the changes in mRNAs induced by genistein, and fulvestrant in the absence of genistein had no effects (Fig. 4). The increase in C/EBPβ mRNA induced by genistein was slightly but significantly attenuated by fulvestrant, indicating that at least a component of this response is mediated through the classical ER pathway. Visual inspection of the cells, including staining of the lipid content using Oil Red O, indicating that fulvestrant had no effect on the genistein‐induced change in cell appearance (data not shown). Thus, it appears that these effects of genistein are independent of binding to ERs.

**Figure 5:**
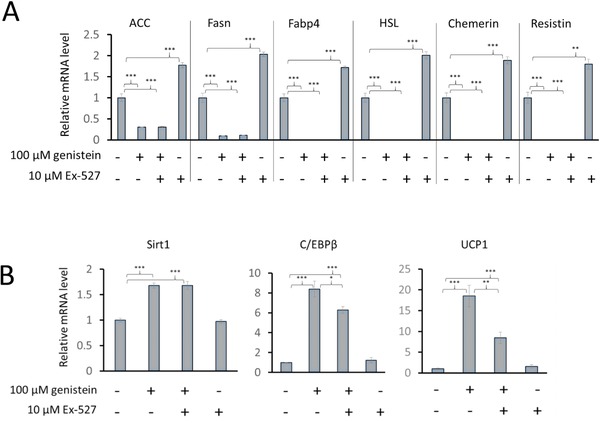
The effect in 3T3‐L1 cells of the Sirt1 inhibitor Ex‐527 on responses to genistein of mRNAs corresponding to genes expressed at high levels in white adipocytes (Panel A) or genes that play a role in stimulating the development of brown/beige adipocytes or that are expressed preferentially in brown/beige compared with white adipocytes (Panel B). Genistein (100 μM), with or without Ex‐527 (10 μM), as indicated, was applied on induction of differentiation (day 0) and measurements were made on day 12. Data are expressed relative to the reference genes *Topo1* and *NONO* and normalised to control. Values are mean ± SEM for *n* = 3. Statistical analysis was by one‐way ANOVA followed by Bonferroni’ multiple comparisons test. *p ≤ 0.05, **p ≤ 0.01, ***p ≤ 0.001.

SIRT1 has pleiotropic roles in protecting against a variety of features of ageing, including metabolic disease, and overexpression in 3T3‐L1 cells was reported to increase PPAR‐Υ deactylation and induce a gene expression profile characteristic of brown adipocytes 25. Thus, we sought to determine if SIRT1 is a hub through which the other effects of genistein on gene expression we observed are mediated by measuring the same mRNAs after including the SIRT1 inhibitor Ex‐527 in the culture medium along with 100 μM genistein. We found that Ex‐527 did not affect the response to genistein of any of the tested genes that characterize white adipocytes (Fig. 5). However, the expression of this panel of genes was increased significantly when the cells were induced to differentiate in the presence of Ex‐527 alone, which indicates the involvement of Sirt1 activity in regulating the process of lipogenesis. Importantly, inhibition of Sirt1 activity reduced the genistein‐mediated increase in UCP1 and C/EBPβ mRNAs, whereas they were unaffected by Sirt1 inhibition alone (Fig. 5) revealing that at least a component of these responses is via Sirt1.

## 4 Discussion

The present data show that application of the isoflavone genistein during the differentiation process in the well‐established 3T3‐L1 adipocyte cell line model promotes the acquisition of features of brown rather than white adipocytes. Classical brown adipose tissue, such as observed in rodents, human neonates and, more recently, adult humans derives from cells that appear to be of distinct embryological origin to white adipose tissue. Brown adipocytes, like skeletal myoblasts, derive from Myf5‐positive precursors, whereas beige adipocytes appear to derive from a Myf5‐negative lineage, which is generally, though not exclusively, a feature also of white adipocytes.[9, 10, 11, 28] Cells with a lipid distribution, metabolic phenotype and molecular signature more similar to brown than white adipose tissue have been observed in depots of white adipose tissue 11, leading to use of the term beige adipose tissue to describe such depots, and hence the term beige adipocyte. Given that 3T3‐L1 is a fibroblast cell line (thus of Myf5‐negative origin), the cells of the brown‐like phenotype and profile we observe after treatment with genistein should be considered “beige” rather than brown. The features induced by genistein in this work that reveal the acquisition of features of beige rather than white adipocytes comprise: i) a change in lipid droplet distribution from the large droplets typical of white adipose tissue to the multilocular pattern of smaller droplets observed in brown/beige adipose tissue; ii) a change in the expression profiles of genes in three categories – markers of white adipocytes (reduced), mediators of brown/beige adipocyte differentiation (increased) and markers of beige adipocytes (increased); iii) a change in mitochondrial oxygen consumption consistent with greater respiratory uncoupling.

Reports including increased supraclavicular BAT volume and activity detected using 2‐deoxy‐2‐[^18^F]fluoro‐D‐glucose ([^18^F]FDG) PET/CT scanning in human subjects 2, 3 and associated reduction in body fat mass achieved in response to cold acclimation in humans 3, support an argument that may be realistic to target nutritional strategies to promote the acquisition of beige adipose tissue. Thus, the results of the current in vitro work encourage in vivo studies in rodents or humans to determine if such effects translate to whole organism physiology. In this context, a pertinent question is whether or not sufficient local concentrations of genistein are achievable. Plasma concentrations of genistein measured in cohorts with diets rich in isoflavones are typically in the order of 0.1–1 μM e.g. 29, 30, 31. Intervention studies, including our own work 32, 33, 34, have achieved plasma concentrations of around 5 μM with doses in the order of 10 mg. Doses as high as 500 mg /day have been given safely 35, 36. Extrapolation suggests that such intakes could result in plasma concentrations of up to 250 μM, which is well in excess of the extracellular concertation of 100 μM we found to be effective in promoting the beige adipocyte profile. Moreover, it is well‐recognised that effective concentrations of dietary agents that achieve efficacy in cell culture models are generally much higher than concentrations that can induce parallel effects in vivo, and the lipophilic nature of the compound, combined with an exposure period that will be much longer than can be used in cell culture, makes accumulation at concentrations in fatty tissues that exceed those in plasma very likely.

Our finding that genistein effects these changes in adipocytes grown in vitro reveals a direct action on adipose tissue and, importantly, provides a model in which to begin to probe the underlying mediating molecular pathways. We show that the effect of genistein is largely unaffected by inhibition of the classical ER pathway. Thus, despite the fact that gensistein has been shown to have action mediated through the ER, these particular actions appear to be through other pathways, which may include the G‐protein coupled estrogen receptor (GPER) 37, through which genistein has been shown to act (e.g. 38).

An important finding was that the presence of the Sirt1 inhibitor Ex‐527 curtailed substantially the effect of genistein to increase UCP1 mRNA, indicating that activation of Sirt1 may be pivotal in the action of genistein to effect the switch to the uncoupled mitochondrial phenotype that distinguishes brown adipocytes from white adipocytes and is the core of the mechanism through which excess energy is dissipated by brown adipose tissue. Constituent with this observation, Sirt1 overexpression increased PPAR‐Υ deactylation in 3T3‐L1 cells and induced a gene expression profile characteristic of brown adipocytes 25. These actions of Sirt1 to promote the acquisition of brown adipose tissue, for which we here identify a dietary stimulator, are likely to be one of the pleiotropic actions of Sirt1 responsible for its well‐documented effects to extend healthspan.

A pertinent question related to our findings is if these putative beige adipocytes derive from a precursor cell population or through transdifferentiation of induced white adipocytes. We replicated similar responses using different treatment regimes, including application of genistein at later points after induction of differentiation (data not shown). However, these observations do not resolve the issue, since it remains entirely possible that, despite the abundance of cells with features of white adipocytes in the culture when genistein was introduced, the cells characterised by the features of beige adipocytes arose from precursor cells, rather than through transdifferentiation of this established white adipocyte population. Thus, our current work is focused on introducing specific genetic markers that are trackable through the different pathways of differentiation to resolve this question. While efficacy of genistein to induce metabolic benefits through this process is not determined by which of the two mechanisms applies, better understanding of the process will inform work to identify dietary or pharmaceutical agents that are more efficacious in inducing these changes.

A new study with the goal of identifying small molecules that have the ability to achieve metabolic conversion of white to brown adipocytes, using a human pluripotent stem cell‐derived adipocyte model, found the most efficacious agent to be an inhibitor of Janus Kinase (JAK) activity 39, uncovering for the first time a role for the JAK‐STAT pathways in control of this process. Like the present work, these findings do not exclude either transdifferentiation of while adipocytes or *de novo* induction of precursors of brown/beige adipocytes as being the process through which the transformation was achieved. However, like our own work, this important study reveals molecular pathways to target in future in vivo work aimed to translate the findings into achieving metabolic benefit in humans, and thus contributing to reducing the burden of obesity and metabolic disease.

In summary, we show that genistein effects in adipocytes changes the appearance, gene expression profile and mitochondrial function that all point to a switch from white to brown or beige phenotype. Importantly we show these effects in vitro, which reveals a direct action of the compound on adipose tissue. Thus, in vitro models, such as the 3T3‐L1 cell line used in the current work as well as stimulated human pluripotent stem cell‐derived mesenchymal progenitor cells 39, provide a valuable tool to probe in more detail the underlying mediating molecular pathways. Such work may identify novel molecular targets for either dietary or pharmacological intervention to promote the development of beige adipose tissue in humans and hence improve metabolic health.

*S.A.A. contributed to experimental design and performed cell culture experiments, nucleic acid and protein extraction, molecular analyses and data preparation and analysis. L.A.W. provided supervision to S.A.A. and contributed to experimental design and preparation of the manuscript. SM performed measurements of mitochondrial function and contributed to preparation of the manuscript. G.A. established and optimised the 3T3 cell culture model and methods for analysis. J.E.H. provided supervision to S.A.A. and contributed to experimental design. D.F. was responsible for overall design of the study, supervision of S.A.A., and preparation of the manuscript*.

*The authors have declared no conflict of interest*.
